# 

**Published:** 1902-08-02

**Authors:** 


### NOTICE TO CORRESPONDENTS.

All MSS., Letters, Books for Review, and other matters intended for the Editor should be addressed The Editor, "The Hospital," The Hospital Building, 28 & 29 Southampton Street, Strand, W.C.

The Editor cannot undertake to return rejected MS., even when accompanied by stamped directed envelope.

**Notice to Advertisers and Subscribers.**

All Advertisements, Orders for Copies of The Hospital, and Business Communications should be addressed to **THE MANAGER, (Not to the Editor)**, The Hospital, 28 & 29 Southampton Street, Strand, London, W.C.

The Cost of Subscription, post free, is as follows.—Per week, 2½d.; per quarter, 2s. 8d.; per half-year, 5s. 3d.; per annum, 10s. 6d. Foreign:—Per annum, 15s. 2d., payable, in all cases, in advance.

**NOTICE.—Vol. XXXI. of "The Hospital" (October 1901, to March 1902), in handsome cloth binding is now on sale at the office of this Journal, price, 7s. 6d. Cases for binding the Numbers contained in this Volume 1s. 6d. Index to Vol. XXXI., 2½d., post free.**

**The Monthly Part for August is now ready. Price 6d., by post 8d.**

### BOOKS RECEIVED.

Ash and Co., 42 Southwark Street, London, S.E.

"Nursing Guide and Register of Nurses, Guy's Hospital." 1902.

Burlington House, Cambridge.

"University Correspondence College Calendar, 1901–1902."

Baillière, Tindall and Cox.

"Aids to Practical Dispensing." By C. J. S. Thompson.

Longmans, Green and Co.

"The Thompson Yates Laboratories Report." Edited by Robert Boyce and C. S. Sherrington. Vol. IV., Part II. 1902.

John Bale, Sons and Danielsson.

"Some Points in Practical Surgery." By Reginald Harrison, F.R.C.S.

Curtis and Beamish, Coventry.

"Report on the Health of the City of Coventry for 1901." By E. Hugh Snell, M.D., B.Sc.Lond., F.R.S.Ed., M.O.H.

Mackie and Co., Warrington.

"Report on the Health of the County Borough of Warrington for 1901." By J. Guest Gornall, M.A., M.B., D.P.H., M.O.H.

Eyre and Spottiswoode.

"Report on Voluntary Organisations in Aid of the Sick and Wounded during the South African War." By the Central British Red Cross Committee.

### Scraps and Gleanings.

The Rochdale Infirmary has benefited to the extent of £1,000 under the will of the late Mr. C. Barraclough, of Rochdale.

The total cost of building the new Passmore-Edwards Hospital at East Ham and acquiring the land amounted to £9,300, towards which Mr Passmore-Edwards contributed £5,000 and £3,500 was collected in East Ham and adjoining districts. The cost of the furniture, fittings, and general equipments, £1,200, has been generously met by two friends of the hospital, which provides accommodation for 20 patients.

### The Student of Medicine.

### Appointments.

**J. C. Maxwell Bailey,** M.B.Lond., M.R.C.S., L.R.C.P., appointed House Surgeon to the West London Hospital. **H. Wansey Bayly,** M.R.C.S., L.R.C.P.Lond., appointed House Physician to the West London Hospital. **T. Black,** M.B., appointed District Medical Officer of the North Witchford Union. **Edwin Bramwell,** M.B., C.M.Edin., M.R.C.P.Lond., appointed Assistant Physician to the Leith Hospital. **R. J. Collie,** M.D., C.M.Aberd., appointed Assistant Medical Officer to the School Board for London. **Thomas Glasbrook Davies,** appointed Medical Officer for the No. 8 District of the Swansea Union. **Arthur Evans,** M.S, M.D.Lond., F.R.C.S.Eng., appointed Surgeon to Out-patients at the Seamen's Branch Hospital in the Royal Albert Dock. **A. Hopewell-Smith,** L.R.C.P.Lond., M.R.C.S., L.D.S.Eng., appointed Lecturer on Dental Anatomy and Physiology at the Royal Dental Hospital of London. **William Hunter,** M.D.Edin., F.R.C.P.Lond., appointed Physician to the London Fever Hospital. **Edwin Matthew,** M.A., M.B., C.M.Edin., appointed Assistant Physician to the Leith Hospital. **J. Gawlor Murray,** L.R.C.P., L.R.C.S.Edin., L.F.P.S.Glasg., appointed Certifying Factory Surgeon for the Scarborough District. **Henry William Pepper,** M.B., B.Ch., M.R.C.S., L.R.C.P., appointed District Medical Officer to the Parish of Birmingham. **Aubrey H. Pollock,** M.R.C.S., L.R.C.P., appointed House Surgeon to the West London Hospital. **Hugh Mallinson Rigby,** M.B., M.S.Lond., F.R.C.S.Eng., appointed Assistant Surgeon to the London Hospital. **C. J. Thomas,** B.Sc., M.B.Lond., appointed Assistant Medical Officer to the School Board for London. **C. J. Toye,** M.D., B.S.Lond., appointed Medical Officer of Health to the Northern Urban District Council. **Penrose Williams,** M.R.C.S., L.R.C.P., appointed Medical Officer to the Bridgwater Workhouse.

### "The Hospital" Institutional Library.

"Hospitals and Asylums of the World." 4 Vols., with a Portfolio of Plans, complete £12 12s.
"Burdett's Hospitals and Charities, 1902." 5s.
"Cottage Hospitals, General, Fever, and Convalescent." 10s. 6d.
"Hospital Expenditure: The Commissariat." 2s. 6d.
"A Legal Handbook for the Use of Hospital Authorities." 2s. 6d.
"The Cottage Hospital Case Book or Register of Patients." 10s. and 12s. 6d.
"The Furnishing and Appliances of a Cottage Hospital." 6d.
All these are published by the Scientific Press, Ltd., and may be obtained through any bookseller, or direct from the publishers, 28 and 29 Southampton Street, Strand, London, W.C.

